# Unusual chromosomal polymorphism of the common shrew, *Sorex araneus* L., in southern Belarus

**DOI:** 10.3897/CompCytogen.v15.i2.63084

**Published:** 2021-06-04

**Authors:** Iryna A. Kryshchuk, Victor N. Orlov, Elena V. Cherepanova, Yury M. Borisov

**Affiliations:** 1 Scientific and Practical Center for Bioresources, National Academy of Sciences of Belarus, Akademicheskaya St. 27, 220072 Minsk, Belarus National Academy of Sciences of Belarus Minsk Belarus; 2 Severtsov Institute of Ecology and Evolution, Russian Academy of Scences, Leninskij Prosp. 33, 119071 Moscow, Russia Russian Academy of Scences Moscow Russia

**Keywords:** Acrocentric morph, chromosomal race, Hardy-Weinberg ratio, dihybrid and trihybrid segregation

## Abstract

Analysis of the frequency of karyotypes and chromosomal rearrangements in the distributional ranges of four metacentric races of *Sorex araneus* Linnaeus, 1758 has revealed features that are not typical for polymorphic populations of this species. The frequency of the acrocentric karyotype and heterozygotes for fusion of acrocentric chromosomes turned out to be significantly higher than expected in case of random crossing. As an explanation for the unusual polymorphism, it has been suggested that metacentric races may hybridize with acrocentric populations that remained from the ancient chromosomal form.

## Introduction

The polymorphic part of the karyotype of the common shrew, *Sorex araneus* Linnaeus, 1758, is represented by 12 pairs of acrocentric chromosomes (*g*, *h*, *i*, *j*, *k*, *l*, *m*, *n*, *o*, *p*, *q*, and *r*) that have a capacity to form new arm combinations in metacentric chromosomes. The recognition, naming, and systematics of populations with different karyotypes has long been an important issue in the common shrew (Bulatova et al. 2019).

**Figure 1.**
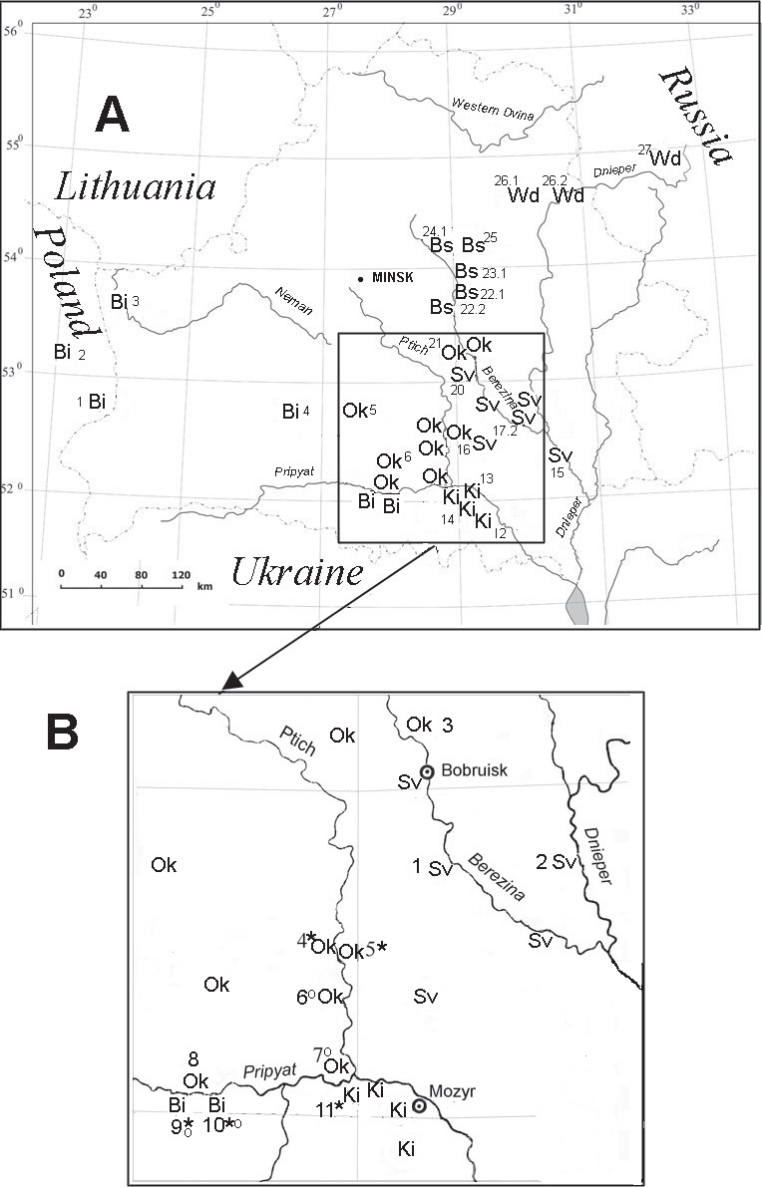
**A** Collection sites and distribution of chromosomal races *Sorex araneus* races in Belarus. Short abbreviations of races and their numbers indicate samples according to previously published (Borisov et al. 2016). Chromosomal races: Ki – Kiev; Sv – Svetlogorsk; Bi – Białowieża; Ok – Oktiabrskiy, Wd – West Dvina, Bs – Borisov **B** enlargement showing the sampling area. Site number as in Table 1, * – marked numbers of samples with single hybrids, o – marked numbers of samples with single karyotypes (*hk*) of the Borisov race.

Three races of the common shrew with race-specific metacentrics, West Dvina (*gm*, *hk*, *ip*, *no*, *qr*), Białowieża (*g/r*, *h/n*, *ik*, *m/p*), and Kiev (*g/m*, *hi*, *k/o*) races, have been previously described near the northern, western, and southern borders of Belarus (Fredga and Nawrin 1977; Mishta et al. 1994; Bulatova et al. 2002). An investigation of karyotypes in Belarus showed that some of race-specific metacentrics disappear from the polymorphic populations, and the frequency of the remaining ones decreases from 0.7–1.0 to 0.4–0.5 (Borisov et al. 2010, 2014). To describe this complex polymorphism, it was proposed to distinguish three new chromosomal races that differ in the set of metacentrics from the previously described chromosomal races: new race Borisov (*g/m*, *h/k*, *i*, *n/o*, *p*, *q/r*) from the West Dvina race, new race Oktiabrskiy (*g*, *r*, *h/n*, *i/k*, *m*, *p*, *o*, *q*) from the Białowieża race, and new race Svetlogorsk (*g*, *m*, *h/i*, *k/o*, *n*, *p*, *q*, *r*) from the Kiev race (Fig. 1a) (Borisov et al. 2010, 2017).

Two hypotheses of the origin of chromosomal polymorphism in populations of the common shrew in Belarus were proposed and discussed (Borisov et al. 2014). The first explains chromosomal polymorphism in Belarus by hybridization between metacentric races and ancient acrocentric populations characterized by 10 pairs of acrocentric chromosomes (*g*, *h*, *i*, *k*, *m*, *n*, *o*, *p*, *q*, *r*) that previously existed on the territory of Belarus. The acrocentric karyotypes (morphs) were revealed in some polymorphic populations of the common shrew in southern Belarus (Borisov et al. 2014). However, we have no proof of this hypothesis. Therefore, we didn’t rule out the possibility that the origin of the Oktiabrskiy and Svetlogorsk races resulted from hybridization of the Białowieża and Kiev races. In wide hybrid zones, an “acrocentric peak” may occur, “that is a source of selection against the monobrachial hybrids in hybrid zones of chromosomal races with metacentrics and hence results in an increase in frequency of acrocentric morphs” (Searle 1986, p. 278). The Oxford–Hermitage hybrid zone in England and the Drnholec–Łęgucki Młyn hybrid zone in Poland are the best examples of these types of wide hybrid zones (Fedyk et al. 2019).

To investigate the causes of chromosomal polymorphism in the common shrew in southern Belarus, we collected additional samples in peripheral parts of the ranges of four chromosomal races (Fig. 1a). We planned to check whether the frequency of karyotypes in polymorphic populations corresponds to random crossing or deviates from it to obtain evidence for one of the two hypotheses under consideration for the origin of chromosomal polymorphism in southern Belarus.

## Material and methods

The study area is a mosaic of forest and meadow biotopes. Animals were captured in 11 sites in the area between the Berezina, Ptich, and Pripyat rivers (Gomel and Mogilev regions) in July–September, 2017–2018 (Fig. 1b). In total, 279 individuals from 11 sites have been analyzed in this survey (Table 1).

**Table 1.** Collection sites, karyotypes of the individual common shrews, and polymorphic chromosome races in the southern Belarus. In the karyotype characteristics, only the variable autosomal arms are included (*g*–*r*). These arms can be presented in a dissociated state as individual acrocentric autosomes (e.g. *h*, *i*, *k*, *o*) or as components of metacentric autosomes (e.g. *hi*, *ko*). The presence of heterozygous karyotypes is indicated by a slash between the two arms, e.g. *h/i*, *k/o* (follows Bulatova et al. 2019). Chromosomal races: Ki – Kiev; Sv – Svetlogorsk; Bi – Białowieża; Ok – Oktiabrskiy, Bs – Borisov, H – hybrids.

No.	Collection site	Latitude, Longitude	Short abbreviation of races	2NA	Karyotype	Number of shrews	
						New data	Borisov et al. (2017)
Polymorphic Svetlogorsk (Sv) race (*h/i*, *k/o*)							
1	Parichi	52°48'04"N, 29°25'58"E	Sv	28	*g*, *h*, *i*, *k*, *m*, *n*, *o*, *p*, *q*, *r*	–	3
			Sv	26	*g*, *h/i*, *k/o*, *m*, *n*, *p*, *q*, *r*	–	9
			Sv			–	(12)
2	Zhlobin	52°50'32"N, 29°45'35"E	Sv	28	*g*, *h*, *i*, *k*, *m*, *n*, *o*, *p*, *q*, *r*	–	1
			Sv	26	*g*, *h/i*, *k/o*, *m*, *n*, *p*, *q*, *r*	–	8
						–	(9)
Polymorphic Oktiabrskiy (Ok) race (*h/n*, *i/k*)							
3	Lyubonichi	53°15’19N, 29°10’21E	Ok	28	*g*, *h*, *i*, *k*, *m*, *n*, *o*, *p*, *q*,	2	2
			Ok	25	*g*, *hn*, *i/k*, *m*, *o*, *p*, *q*, *r*	4	2
			Ok	25	*g*, *h/n*, *ik*, *m*, *o*, *p*, *q*, *r*	1	2
			Ok	26	*g*, *h/n*, *i/k*, *m*, *o*, *p*, *q*, *r*	3	5
			Ok	27	*g*, *h/n*, *i*, *k*, *m*, *o*, *p*, *q*, *r*	2	1
			Ok	27	*g*, *h*, *i/k*, *m*, *n*, *o*, *p*, *q*, *r*	3	–
			Ok			(15)	(12)
4	Rozhanov Oktiabrskiy	52°35'51"N, 28°45'08"E	Ok	28	*g*, *h*, *i*, *k*, *m*, *n*, *o*, *p*, *q*, *r*	5	3
			Ok	26	*g*, *h/n*, *i/k*, *m*, *o*, *p*, *q*, *r*	19	3
			H	25	*g*, *h/n*, *i*, *ko*, *m*, *p*, *q*, *r*	2	–
						(26)	(6)
5	Zatishie (Oktiabrskiy)	52°34'26"N, 28°44'37"E	Ok	28	*g*, *h*, *i*, *k*, *m*, *n*, *o*, *p*, *q*, *r*	2	–
			Ok	26	*g*, *h/n*, *i/k*, *m*, *o*, *p*, *q*, *r*	15	–
			Sv	26	*g*, *hi*, *k*, *m*, *n*, *o*, *p*, *q*, *r*	1	2
						(18)	(2)
6	Luchicy	52°27'16"N, 28°48'35"E	Ok	28	*g*, *h*, *i*, *k*, *m*, *n*, *o*, *p*, *q*, *r*	4	2
			Ok	26	*g*, *h/n*, *i/k*, *m*, *o*, *p*, *q*, *r*	8	–
			Bs	27	*g*, *h/k*, *i*, *m*, *n*, *o*, *p*, *q*, *r*	1	1
						(13)	(3)
7	Konkovichi	52°9'22"N, 28°43'30"E	Ok	28	*g*, *h*, *i*, *k*, *m*, *n*, *o*, *p*, *q*, *r*	6	4
			Ok	26	*g*, *h/n*, *i/k*, *m*, *o*, *p*, *q*, *r*	21	2
			Ok	25	*g*, *hn*, *i/k*, *m*, *o*, *p*, *q*, *r*	7	–
			Ok	24	*g*, *hn*, *ik*, *m*, *o*, *p*, *q*, *r*	9	–
			Bs	27	*g*, *h/k*, *i*, *m*, *n*, *o*, *p*, *q*, *r*	–	1
						(43)	(7)
8	Borki	52°05'50"N, 27°49'19"E	Ok	28	*g*, *h*, *i*, *k*, *m*, *n*, *o*, *p*, *q*, *r*	–	1
			Ok	25	*g*, *r*, *hn*, *i/k*, *m*, *o*, *p*, *q*	–	1
			Ok	27	*g*, *m*, *h/n*, *i*, *k*, *o*, *p*, *q*, *r*	–	1
			Ok	27	*g*, *r*, *h*, *n*, *i/k*, *m*, *o*, *p*, *q*	–	1
						–	(4)
Polymorphic Białowieża (Bi) race ([*gr* or *mp*], *hn*, *ik*)							
9	Turov	52°04'15"N, 27°45'48"E	Bi	28	*g*, *h*, *i*, *k*, *m*, *n*, *o*, *p*, *q*, *r*	–	4
			Bi	25	*g*, *h/n*, *i/k*, *m/p*, *o*, *q*, *r*	27	1
			H	26	*g*, *h*, *ik/ko*, *m*, *n*, *p*, *q*, *r*	–	1
			Bs	27	*g*, *h/k*, *i*, *m*, *n*, *o*, *p*, *q*, *r*	2	2
						(30)	(7)
10	Khvoyensk	52°2'11"N, 27°56'40"E	Bi	28	*g*, *h*, *i*, *k*, *m*, *n*, *o*, *p*, *q*, *r*	10	–
			Bi	25	*g/r*, *h/n*, *i/k*, *m*, *o*, *p*, *q*	8	10
			Bi	25	*g*, *hn*, *i/k*, *m*, *o*, *p*, *q*, *r*	3	3
			Bi	26	*g*, *h/n*, *i/k*, *m*, *o*, *p*, *q*, *r*	1	3
			Bi	24	*g*, *hn*, *ik*, *m*, *o*, *p*, *q*, *r*	1	2
			H	26	*g*, *h/n*, *k/o*, *m*, *i*, *p*, *q*, *r*	2	–
			Bs	27	*g*, *h/k*, *i*, *m*, *n*, *o*, *p*, *q*, *r*	2	–
						(27)	(18)
Polymorphic Kiev (Ki) race (*g/m*, *h/i*, *k/o*)							
11	Skrygalov	52°03'20"N, 28°49'10"E	Ki	28	*g*, *h*, *i*, *k*, *m*, *n*, *o*, *p*, *q*, *r*	1	–
			Ki	25	*g/m*, *h/i*, *k/o*, *n*, *p*, *q*, *r*	25	–
			H	26	*g*, *h*, *ik/ko*, *m*, *n*, *p*, *q*, *r*	1	–
						(27)	–
						199	80

Chromosome mounts were prepared from bone marrow and spleen cells after a routine technique with colchicine treatment (Ford and Hamerton 1956). Individual chromosome identification was carried out after G-band staining procedure with trypsin (Seabright 1971) in accordance with the international common shrew chromosome nomenclature (Searle et al. 1991).

For statistical procedures, we proceeded from a single, two- and three- locus model with a codominant type of inheritance. The calculations were based on the matrix of individual karyotypes (Table 1). The frequency of karyotypes in the polymorphic populations of the Oktiabrskiy (*h/n*, *i/k*) and Svetlogorsk (*h/i*, *k/o*) races determine by combinations of four types of gametes (dihybrid segregation) and in the Białowieża ([*g/r* or *m/p*], *h/n*, *i/k*) and Kiev (*g/m*, *h/i*, *k/o*) races by combinations of eight types of gametes (trihybrid segregation).

According to the principles of Mendelian inheritance, nine different karyotypes are theoretically possible in polymorphic populations of the Oktiabrskiy or Svetlogorsk races (dihybrid segregation): (*hn*, *ik*), (*hn*, *i*, *k*), (*h*, *n*, *ik*), (*h*, *n*, *i*, *k*), (*hn*, *i/k*), (*h*, *n*, *i/k*), (*h/n*, *ik*), (*h/n*, *i*, *k*), and (*h/n*, *i/k*). In the case of a random combination of gametes and the absence of selection, the expected frequencies of genotypes (e. g. karyotypes) are constant, their values are given in genetic reference books. The expected frequency of the acrocentric (*h*, *n*, *i*, *k*) and homozygous (*hn*, *ik*) karyotypes should be 1/16 (0.0625), and heterozygous one (*h/n*, *i/k*) – ¼ (0.25). Similarly, in populations polymorphic by three metacentrics (Białowieża and Kiev races), 27 genotypes are theoretically possible (trihybrid segregation). The expected frequency of the acrocentric ([*g*, *r* or *m*, *p*] *h*, *n*, *i*, *k*) and homozygous ([*gr* or *mp*], *hn*, *ik*) karyotypes should be 1/64 (0.0156), and heterozygous one ([*g/r* or *m/p*], *h/n*, *i/k*) – 1/8 (0.125).

The expected frequency of homozygotes and heterozygotes was estimated by the Hardy–Weinberg equation: *p*^2^+2*pq*+*q*^2^=1, where *p*^2^ is the proportion of homozygotes for one of the alleles (e. g. *hn*), *p* is the frequency of this allele, 2*pq* is the proportion of heterozygotes (*h/n*), *q*^2^ is the proportion of homozygotes for the alternative allele (*h*, *n*), and *q* is the frequency of the corresponding allele. We used a variant of the Hardy–Weinberg equation for small samples (Li 1976).

## Results

The distribution of the four chromosomal races in southern Belarus is shown in Fig. 1a. In southwestern Belarus, between the Pripyat and Neman Rivers, the complete set of race-specific metacentrics for the Białowieża race was known from populations eastward to Ganzevichi (26°25'E) (Fig. 1). Only two metacentrics, *hn* and *ik*, (Oktiabrskiy race) were found in populations 100 km eastward up to the Ptich and Pripyat Rivers (Fig. 1b, *4*–*8*), and up to the Berezina river (Fig. 1b, *3*). South of the Pripyat River, five metacentrics of the Białowieża race were found in Turov – Chvoyensk (27°56'E) (Fig. 1b, *9*, *10*).

We found rare hybrids of the Białowieża and Kiev races (recombinants *h/n*, *k/o* and complex heterozygotes *ik/ko*) in two localities of the Białowieża race distribution and in one locality inhabited by the Kiev race along the southern bank of the Pripyat river (Fig. 1b, *9*–*11*). The width of the hybrid zone between the Białowieża and Kiev races can reach 70 kilometers. Three shrews with metacentric *hi* (Svetlogorsk race) and two hybrids of the Oktiabrskiy–Svetlogorsk races (*h/n*, *ko*) were found in localities of the Oktiabrskiy race distribution (Fig. 1b, *4*, *5*). The data allowed us to suppose that the Oktiabrskiy–Svetlogorsk hybrid zone stretches along the Ptich river.

**Figure 2.**
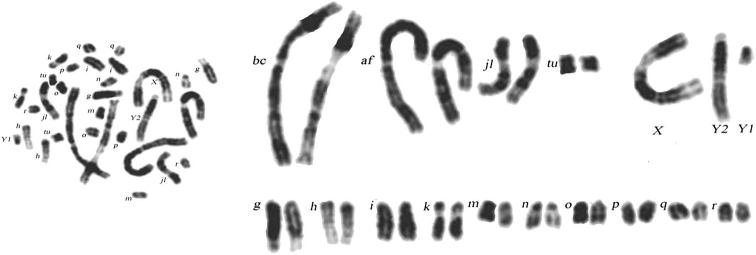
G-banded karyotypes of the acrocentric morph of the common shrew (male) from Konkovichi, Oktiabrskiy race.

**Figure 3.**
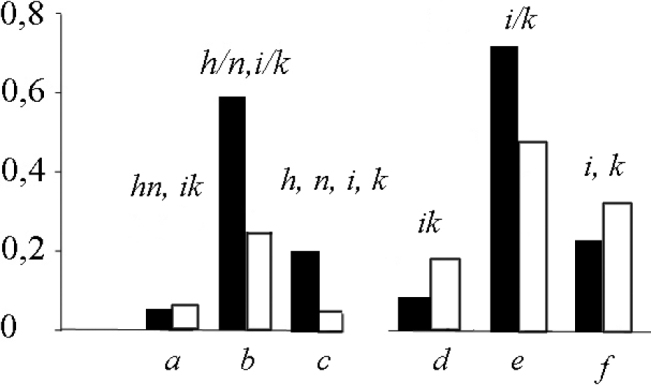
The frequencies of three karyotypes (the acrocentric karyotype and karyotypes homozygous and heterozygous for metacentrics) and homozygotes and heterozygotes for fusion *ik* recorded in five populations of the Oktiabrskiy race (after Table 2). Black bars – observed frequency, white – expected frequency: *a* – homozygous karyotype (*hn*, *ik*), *b* – heterozygous karyotype (*h/n*, *i/k*), *c* – acrocentric karyotype (*h*, *n*, *i*, *k*), *d* – homozygote *ik*, *e* – heterozygote *i/k*, *f* – homozygote *i*, *k*.

The studied samples allowed us to compare the observed and expected frequencies of three karyotypes (morphs) in each chromosomal race, including acrocentric karyotype, homozygous and heterozygous metacentric karyotypes in four races (Tables 2 and 3). Acrocentric karyotypes were found in all studied populations. The chromosomal formula of the acrocentric karyotype (morph): *XX / XY1Y2*, *af*, *bc*, *g*, *h*, *i*, *jl*, *k*, *m*, *n*, *o*, *p*, *q*, *r*, *tu* (Fig. 2). The area of distribution of this acrocentric karyotype in southern Belarus is undoubtedly much wider than the studied area. In all studied chromosomal races, deviations of the frequencies of karyotypes from those expected during dihybrid and trihybrid segregation were revealed. The observed frequencies of the acrocentric karyotype in two races with dihybrid segregation, Oktiabrskiy and Svetlogorsk race, are three times higher than expected and by an order of magnitude then expected in Białowieża race (trihybrid segregation). Only in the sample of the Kiev race, the difference in frequencies is not reliable, probably due to a small sample and a low expected frequency. The observed frequency of karyotype heterozygous for metacentrics in two races with dihybrid segregation are two to three times higher than expected and by an order of magnitude or more than expected in Białowieża and Kiev races (trihybrid segregation). The frequency of the karyotype homozygous for metacentrics *hn* and *ik* is close to that expected in the Oktiabrskiy race (Table 2), but such karyotypes were not found in other races due to small samples and low expected frequency. More clearly, the differences between the observed and expected frequencies in the Oktiabrskiy race are shown in Figure 3.

**Table 2.** The frequency of karyotypes recorded in studied localities of the Oktiabrskiy and Svetlogorsk races; *O* – observed frequency, *E* – expected frequency (after Table 1).

Number of shrews	Karyotypes														
	Acrocentric			Heterozygous			Homozygous			Heterozygous			Homozygous		
	(Ok) *h*, *n*, *i*, *k* or (Sv) *h*, *i*, *k*, *o*			*h/n*, *i/k*			*hn*, *ik*			*h/i*, *k/o*			*hi*, *ko*		
	*O*	*E*	χ^2^	*O*	*E*	χ^2^	*O*	*E*	χ^2^	*O*	*E*	χ^2^	*O*	*E*	χ^2^
Oktiabrskiy race (Ok)															
141	31	8.7	57.2	68	35.2	30.6	9	8.7	NS	–	–	–	–	–	–
	0.220	0.062	***	0.482	0.250	***	0.064	0.062							
Svetlogorsk race (Sv)															
21	4	1.3	5.6	–	–	–	–	–	–	17	5.2	26.8	–	1.3	–
	0.190	0.062	*							0.809	0.250	***		0.062	

Asterisks indicate the significance of differences: * *P* < 0.05, *** *P* < 0.001; NS – not reliable.

Not only the observed frequency of karyotypes with heterozygotes, but also the frequency of heterozygotes for fusion of acrocentric chromosomes significantly exceeded the expected by Hardy-Weinberg, while the observed frequency of homozygotes is less than expected (Table 4, Fig. 3d–f). This ratio of frequencies is maintained both for the two races as a whole and for individual samples. For example, in five samples of the Oktiabrskiy race, the ratios of the observed and expected number of heterozygotes *i/k*, calculated for small samples according to Li, 1976, are 17/13, 22/14, 15/8, 8/6, and 30/24, respectively. For homozygotes *i*, *k*, the ratios are 7/9, 8/12, 2/5, 6/7, 10/13 (Table 1, *3*–*7*).

**Table 3.** The frequency of karyotypes recorded in studied localities of the Białowieża and Kiev races; *O* – observed frequency, *E* – expected frequency (after Table 1).

Number of Shrews	Karyotypes														
	Acrocentric			Heterozygous			Homozygous			Heterozygous			Homozygous		
	(Bi) *g*, *r*, *m*, *p*, *h*, *n*, *i*, *k* or (Ki) *g*, *m*, *h*, *i*, *k*, *o*			[*g/r* or *m/p*] *h/n*, *i/k*			[*gr* or *mp*], *hn*, *ik*			*g/m*, *h/i*, *k/o*			*gm*, *hi*, *ko*		
	*O*	*E*	χ^2^	*O*	*E*	χ^2^	*O*	*E*	χ^2^	*O*	*E*	χ^2^	*O*	*E*	χ^2^
Białowieża race (Bi)															
73	14	1.1	151.2	46	9.1	830	–	1.1	–	–	–	–	–	–	–
	0.192	0.016	***	0.630	0.125	***		0.016							
Kiev race (Ki)															
26	1	1.1	0.009	–	–	–	–	–	–	25	3.25	9.9	–	1.1	
	0.038	0.016	NS							0.961	0.125	***		0.016	

Asterisks indicate the significance of differences: *** *P* < 0.001; NS – not reliable.

**Table 4.** Tests for deviation from the Hardy–Weinberg equilibrium in the highly polymorphic samples of the Oktiabrskiy and Białowieża races; *O* – observed frequency, *E* – expected frequency.

Number of shrews	Homo-, heterozygotes	*O*	*E*	χ^2^
214	*hn*	32	45	14.41 ***
		0.149	0.212	
	*h/n*	133	106	
		0.622	0.497	
	*h*, *n*	49	62	
		0.229	0.291	
	*ik*	15	38	63.30 ***
		0.070	0.177	
	*i/k*	150	104	
		0.701	0.487	
	*i*, *k*	49	72	
		0.229	0.335	

Asterisks indicate the significance of differences: *** *P* < 0.001.

## Discussion

The studied polymorphic populations differ in two features from any other polymorphic populations of the common shrew:

1. The frequency of the acrocentric karyotype and karyotypes heterozygous for metacentrics both in dihybrid and trihybrid segregation turned out to be significantly higher than expected in case of random crossing.
2. The frequency of heterozygotes for fusion of acrocentric chromosomes turned out to be higher than expected according to Hardy-Weinberg in the case of random crossing, and the frequency of homozygotes, on the contrary, is less than expected.

To date, such deviations of the observed frequencies from the expected ones have not been recorded in any polymorphic populations of the common shrew. This frequency of genotypes is not typical for hybrid zones of chromosomal races of the common shrew, in particular, with an “acrocentric peak” (Fedyk et al. 2019). The frequency of karyotypes in polymorphic populations of Belarus has not been previously analyzed, and the frequency of homozygotes and heterozygotes for fusions of acrocentric chromosomes in other polymorphic populations on the territory of Belarus did not differ from those expected according to Hardy-Weinberg (Borisov et al. 2010, 2014).

In the studied hybrid zones of the common shrew, the frequency of simple heterozygotes (CIII) does not differ from that expected in the case of random crossing, and the frequency of more complex heterozygotes is constantly lower than expected even taking into account the Wahlund effect (Orlov et al. 2020). In polymorphic populations and hybrid zones of the common shrew, the advantage of heterozygotes was never observed when their frequency was higher than expected.

Therefore, the polymorphism of the studied populations is not associated with the hybridization of metacentric races (Białowieża–Kiev or Oktiabrskiy–Svetlogorsk). We may suppose that this unusual polymorphism in populations of the common shrew and the origin of the Oktiabrskiy and Svetlogorsk races is caused by the hybridization of metacentric races and the acrocentric population remained in the *ancient chromosomal form*. Two common shrew acrocentric populations without polymorphism were found at the southern border of the species range, in the Alps and the Balkans. These populations were described as chromosomal races Cordon (Hausser et al. 1991) and Pelister (Macholán et al. 1994). Probably, acrocentric populations were previously widespread in Europe and Asia. This is indicated not only by the surviving acrocentric chromosomal races (Cordon and Pelister), but also by the races with a single race-specific metacentric, Baikal, Carlit, Lemland, and Nogat (Bulatova et al. 2019). The possibility of the origin of new chromosomal races of the common shrew by means of the distribution of chromosomal rearrangements in populations with acrocentric chromosomes has been repeatedly noted by several authors (Wójcik 1993; Brünner et al. 2002; Shchipanov and Pavlova 2017).

Therefore, the fitness of the acrocentric karyotype and heterozygous metacentric karyotype may be higher than homozygous metacentric karyotypes in the studied populations. It is the increased fitness that these karyotypes became the factor responsible for the disappearance of some metacentrics, their replacement by acrocentrics, and the origin of new chromosomal races. Previously, a decrease in the frequency of race-specific metacentrics of the West Dvina race from east to west, the Białowieża race from west to east, and race-specific metacentrics of the Kiev race – from south to north in Belarus was shown (Borisov et al. 2010, 2016). Such a change in the frequency of metacentrics may reflect the direction of dispersal of chromosomal races with metacentric chromosomes in Holocene and hybridization with local acrocentric populations.

In some populations of the peripheral parts of the Białowieża and Oktiabrskiy races, there are rare individuals that are heterozygous for the arm combination *hk* with karyotype *g*, *h/k*, *i*, *m*, *n*, *o*, *p*, *q*, *r* (Fig. 1b). This karyotype (morph) was previously described for one shrew as the Turov race from Turov town (Fig. 1b, *9*) (Mishta et al. 2000). As a specific arm combination, *hk* is distributed in ten chromosomal races that are spread from the Baltic Sea to the latitude south of Minsk (Bulatova et al. 2019). The arm combination *hk* is known in the Borisov race with a frequency of 0.833 (Borisov et al. 2010) in 150 km from populations of the Oktiabrskiy race with this morph (Fig. 1a, *22.2*). It is most likely that rare morphs *g*, *h/k*, *i*, *m*, *n*, *o*, *p*, *q*, *r*, enter the Oktiabrskiy race populations from the southern populations of the Borisov race. This is facilitated by the low frequency of race-specific metacentrics, 0.42–0.45, and the high frequency of acrocentric karyotypes in such populations. In our opinion, the Bobruysk race (*g*, *h/i*, *k*, *m*, *n*, *o*, *p*, *q*, *r*) described by two shrews from Bobruysk city (Mishta et al. 2000) is one of the morphs of the Svetlogorsk race In case of random dihybrid segregation, the morph *h/i*, *k*, *o* (Aab) should occur in populations of the Svetlogorsk race with a frequency of 0.125.
